# The copper chelator ammonium tetrathiomolybdate inhibits the progression of experimental endometriosis in TNFR1-deficient mice

**DOI:** 10.1038/s41598-023-37031-1

**Published:** 2023-06-26

**Authors:** Rocío Ayelem Conforti, María Belén Delsouc, Ana Sofia Zabala, Sandra Silvina Vallcaneras, Marilina Casais

**Affiliations:** Facultad de Química, Bioquímica y Farmacia, Universidad Nacional de San Luis (UNSL). Instituto Multidisciplinario de Investigaciones Biológicas de San Luis (IMIBIO-SL-CONICET), D5700HHW San Luis, Argentina

**Keywords:** Reproductive disorders, Metals, Steroid hormones, Molecular biology

## Abstract

The TNF-α/TNFR system is involved in endometriosis (EDT), a gynecologic estrogen-dependent disease. Elevated copper concentrations have also been associated with EDT, even in TNFR1-deficient mice where disease worsening occurs. We aimed to evaluate whether treatment with ammonium tetrathiomolybdate (TM, copper chelator) is beneficial in TNFR1-deficient mice presenting with worsened EDT status. Female C57BL/6 mice were divided into three groups: KO Sham, KO EDT, and KO EDT+TM. TM was administered from the 15th postoperative day, and samples were collected one month after inducing pathology. In peritoneal fluid, copper and estradiol levels were determined by electrothermal atomic absorption spectrometry and electrochemiluminescence, respectively. Lesions were processed for the analysis of cell proliferation (PCNA immunohistochemistry), expression of angiogenic markers (RT-qPCR), and oxidative stress (spectrophotometric methods). We found that EDT increased copper and estradiol levels compared to the KO Sham group, while the TM administration restored the levels of both factors. TM also reduced the volume and weight of the lesions and cell proliferation rate. Besides, TM treatment decreased the number of blood vessels and the *Vegfa*, *Fgf2*, and *Pdgfb* expression. Furthermore, superoxide dismutase and catalase activity decreased, and lipid peroxidation increased. TM administration inhibits EDT progression in TNFR1-deficient mice where the pathology is exacerbated.

## Introduction

Endometriosis (EDT) is a chronic estrogen-dependent disease characterized by the growth of endometrial-like tissue outside the uterus. EDT is currently considered a systemic disease and affects 5–10% of reproductive-age patients in the world^1^. It usually causes severe pelvic pain, heavy bleeding, dysmenorrhea, and subfertility, which can compromise the life quality of patients^2^.

Several studies on EDT have reported the crucial role played by the tumor necrosis factor-alpha (TNF-α) and its receptors (TNFR1 and TNFR2)^3–6^. TNFR1 (TNFRp55) is constitutively expressed in almost all cell types. Its activation leads to pro-inflammatory pathways and programmed cell death. In contrast, TNFR2 (TNFRp75) is expressed on limited cells, such as immune cells, endothelial cells, neural cells, and even some tumor cells^7–9^. This receptor has been shown to mediate signals that promote tissue repair, proliferation, and angiogenesis^9,10^. Cell survival, cell proliferation, and death occur as a balance between the TNFR1 and TNFR2 signaling pathways, demonstrating the significant crosstalk between them^8,10^. In this regard, it has been postulated that in healthy women, endometrial cells do not implant in ectopic sites because TNF-α activates cell death through TNFR1 and inhibits cell proliferation by down-regulating TNFR2^4^. Interestingly, low TNFR1 expression was reported in women with EDT during the late secretory phase, which could favor the survival and growth of menstrual debris outside the uterus^11,12^. In another study in patients, TNFR1 levels were shown to increase and then decrease from minimal to severe stages of the disease. In contrast, the TNFR2 levels and TNF-α increased as EDT worsened^13^. We previously showed that, in TNFR1-deficient mice, a worsening of pathology is observed compared to the EDT progression in wild-type mice^5,6,14^.

Elevated Cu levels have been reported in serum and urine samples from women with EDT^15,16^. This metal has been associated with oxidative stress in this pathology^15^, a process that possibly contributes to the malignant transformation of EDT^17^. Cu homeostasis has also been shown to be dysregulated in many cancers^18^. Cu can act as a pro-angiogenic metal by modulating the expression of vascular endothelial growth factor (VEGF), fibroblast growth factor 2 (FGF-2), and cytokines^19^. In addition, TNF-α secretion can also be regulated by Cu in cancer cells^19^, and it has been reported that TNFR1 activation can mediate the cytotoxic effects of the metal^20,21^. Being a metalloestrogen, Cu can also potentiate the estrogenic action, estradiol-induced tumor cell proliferation^22^ and, at high levels, stimulate the expression of enzymes involved in estradiol synthesis^23^.

There is no cure for EDT; especially, hormone therapy is only partially effective and often has significant side effects, limiting its long-term use^24^. Since none of the current treatments are guaranteed to work in EDT, the pathophysiological implication of the TNF-α/TNFR system highlights the need to study potential new therapies that may be useful for patients with this unbalanced situation^25^. Recently, we demonstrated that the ammonium tetrathiomolybdate (TM) administration to EDT-induced wild-type mice affects the progression of pathology^26^. TM is a fast-absorbing, low-toxicity Cu chelator that was initially developed as a treatment for Wilson's disease^27^ but has also shown anti-proliferative and anti-angiogenic effects in several cancers^28,29^. TM interferes with the activity of numerous cuproenzymes and nuclear factor κB (NF-κB), leading to lower VEGF, FGF-2, and cytokines^28^. Therefore, considering the involvement of the TNF-α/TNFR system and Cu in the EDT, in the present work, we administered TM in mice to evaluate whether copper chelation is beneficial in a TNFR1^−/−^ worsened EDT status.

## Results

### Experimental EDT increased Cu levels in the peritoneal fluid of TNFR1^−/−^ mice, while the administration of TM restored the concentration of this metal

**Figure 1:**
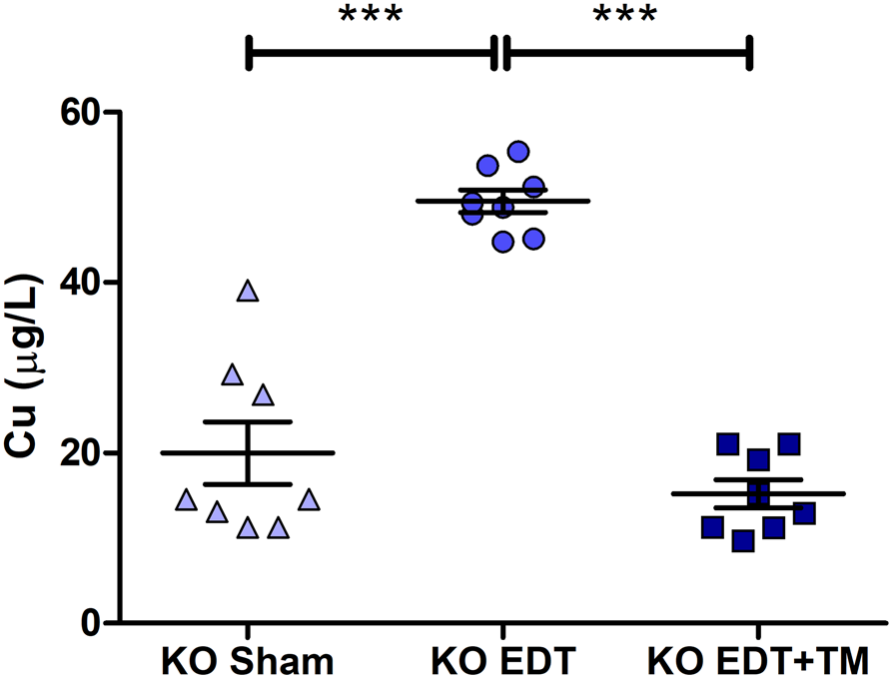
Effect of TM on the Cu concentration in the peritoneal fluid of TNFR1^−/−^ mice. Cu was determined by ETAAS in sham-operated mice (KO Sham; triangles), EDT-induced mice (KO EDT; circles), and TM-treated EDT-induced mice (KO EDT+TM; squares). Results are expressed as mean ± SEM (n = 8 animals/group). One-way ANOVA followed by Tukey's test was used. ***P < 0.001.

Due to the implication of Cu in the EDT progression, we used a chelator and, therefore, we analyzed the levels of the metal in the peritoneal fluid of all experimental animals. On the one hand, the establishment of the pathology (KO EDT group) produced an increase in Cu concentration compared to the KO Sham group (*P* < 0.001; Fig. 1). On the other hand, the administration of TM significantly decreased the concentration of this metal (*P* < 0.001; Fig. 1), reaching values similar to those of the KO Sham group.

### TM affected the development of endometriotic-like lesions in TNFR1-deficient mice

**Figure 2:**
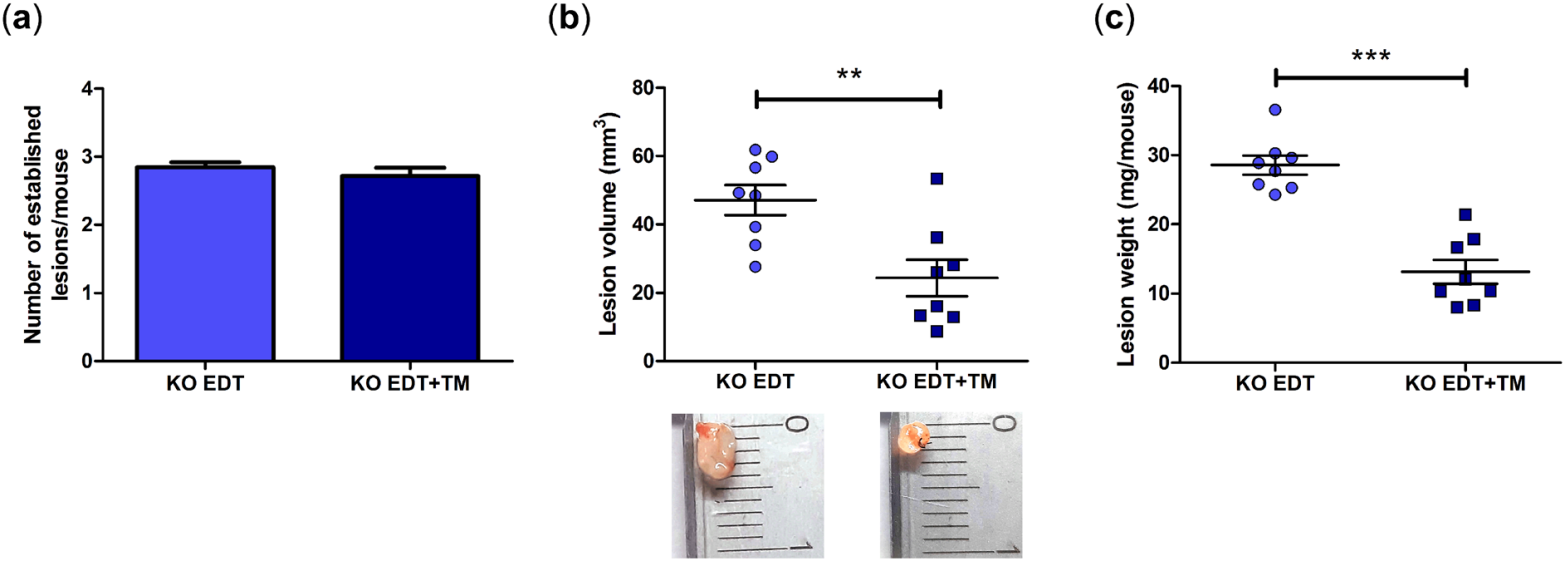
Effect of TM on the development of endometriotic-like lesions in TNFR1^−/−^ mice. The number of established lesions (**a**), their volume (**b**), and their weight (**c**) were evaluated in EDT-induced mice (KO EDT) and TM-treated EDT-induced mice (KO EDT+TM) after one month of inducing the pathology. Representative images of the morphology of endometriotic-like lesions are provided. Results are expressed as mean ± SEM (n = 8 animals/group). KO EDT: circles; KO EDT+TM: squares. Statistical comparisons were made using Student's *t* test. **P < 0.01; ***P < 0.001.

We found that there were no statistically significant differences in the number of lesions established per mouse between experimental groups (Fig. 2a). However, TM administration caused a decrease in both lesion volume (*P* < 0.01, Fig. 2b) and lesion weight (*P* < 0.001, Fig. 2c) compared to the KO EDT group.

### TM modulated estradiol levels in TNFR1-deficient mice

**Figure 3:**
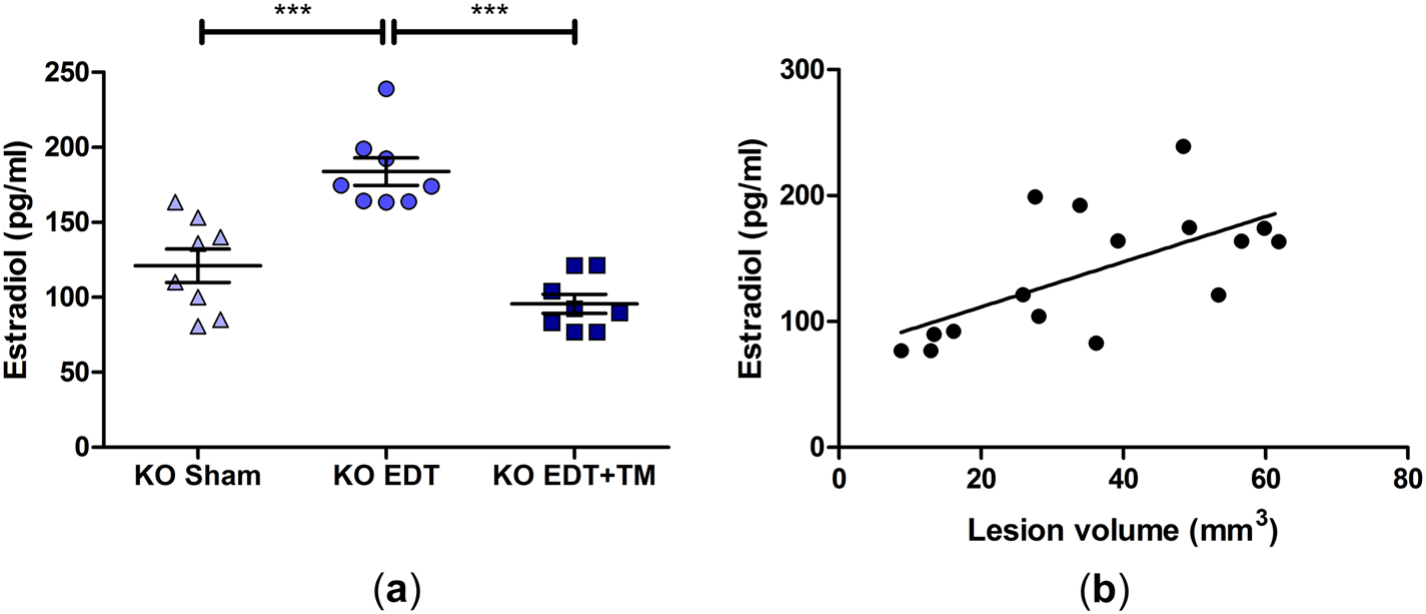
Effect of TM on the estradiol levels in the peritoneal fluid of TNFR1^−/−^ mice. Estradiol was analyzed by ECLIA in sham-operated mice (KO Sham; triangles), EDT-induced mice (KO EDT; circles), and TM-treated EDT-induced mice (KO EDT+TM; squares) (**a**). Results are expressed as mean ± SEM (n = 8 animals/group). One-way ANOVA followed by Tukey's test was used. ***P < 0.001. In addition, a correlation study between the lesion volume and estradiol was performed for the entire group of mice with EDT (n = 16) (**b**). The normality of data was assessed with the Shapiro–Wilk test, and Spearman´s correlation method was applied. *P < 0.05, Spearman r = 0.5548.

Since EDT is an estrogen-dependent disease, estradiol levels in the peritoneal fluid were analyzed. On the one hand, the induction of the pathology produced a significant increase in the levels of this hormone compared to the Sham group (*P* < 0.001; Fig. 3a). On the other hand, TM administration significantly decreased estradiol concentration (*P* < 0.001; Fig. 3a), reaching values similar to those of the KO Sham group. It should be noted that a correlation study demonstrated a moderate positive correlation between the volume of the lesion and estradiol levels (*P* < 0.05; Fig. 3b).

### Treatment with TM decreased the cell proliferation in TNFR1-deficient endometriotic-like lesions

**Figure 4:**
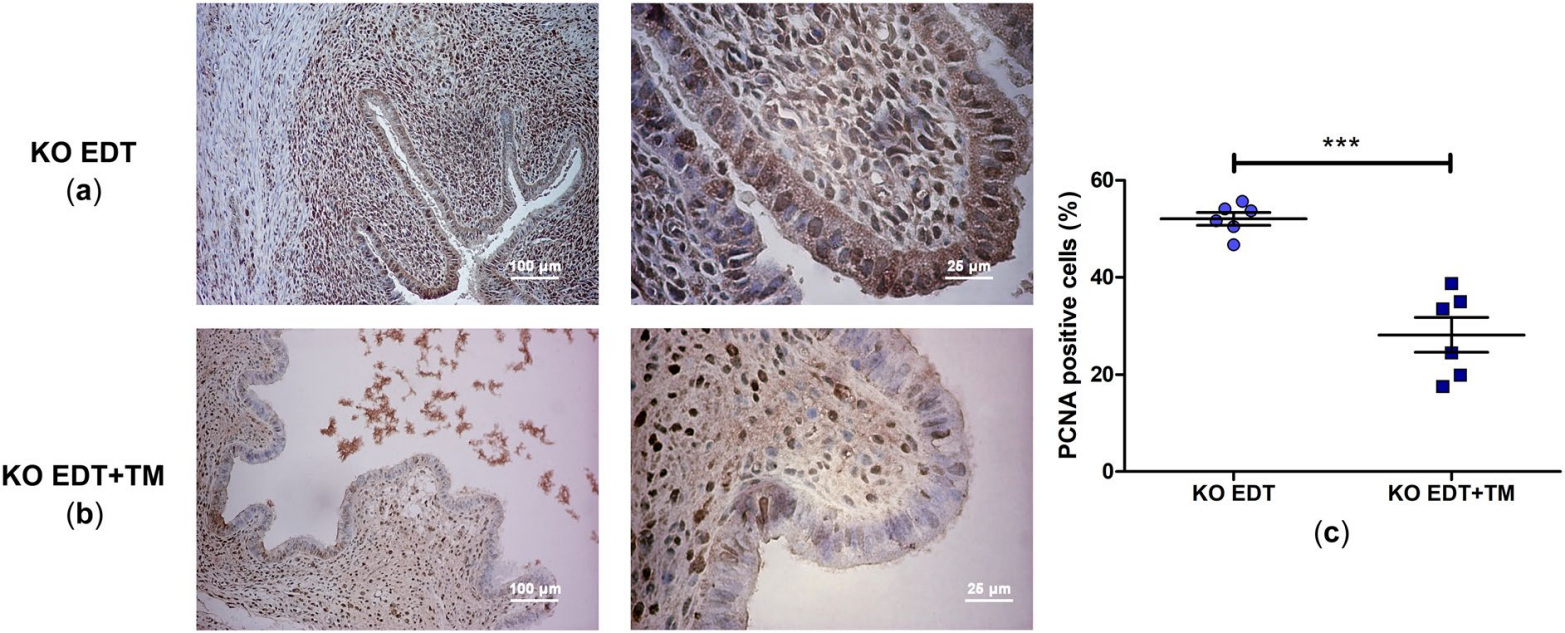
Effect of TM on cell proliferation of endometriotic-like lesions in TNFR1^−/−^ mice. The micrographs show representative histological sections of induced endometriotic-like lesions (magnification: 100 × and 400 ×) in untreated mice (KO EDT) (**a**) and TM-treated mice (KO EDT+TM) (**b**). Scale bar, 100 μm and 25 μm, respectively. The percentage of proliferating cells in endometriotic-like lesions was evaluated by immunohistochemistry for PCNA in both experimental groups (**c**). PCNA-positive cells were identified by the presence of brown nuclear reactivity. Results are expressed as mean ± SEM (n = 6 animals/group). KO EDT: circles; KO EDT+TM: squares. Statistical comparisons were made using Student's *t* test. ***P < 0.001.

Based on the anti-proliferative capacity of TM, we analyzed the PCNA labeling index in lesions sections by immunohistochemistry. Histologically, endometriotic-like lesions from untreated mice showed endometrial glands and abundant stroma, a typical structure consistent with their endometrial origin (Fig. 4a). TM administration notably reduced these EDT histopathological marks (Fig. 4b). This Cu chelator reduced the percentage of PCNA-positive cells (*P* < 0.001; Fig. 4c) compared to the lesions from untreated TNFR1-deficient animals (KO EDT group), in correspondence with the lower weight and volume of the lesions found in the KO EDT+TM group.

### Cu chelation decreased the number of blood vessels and the mRNA expression of *Vegfa*, *Fgf2*, and *Pdgfb* in endometriotic-like lesions

**Figure 5:**
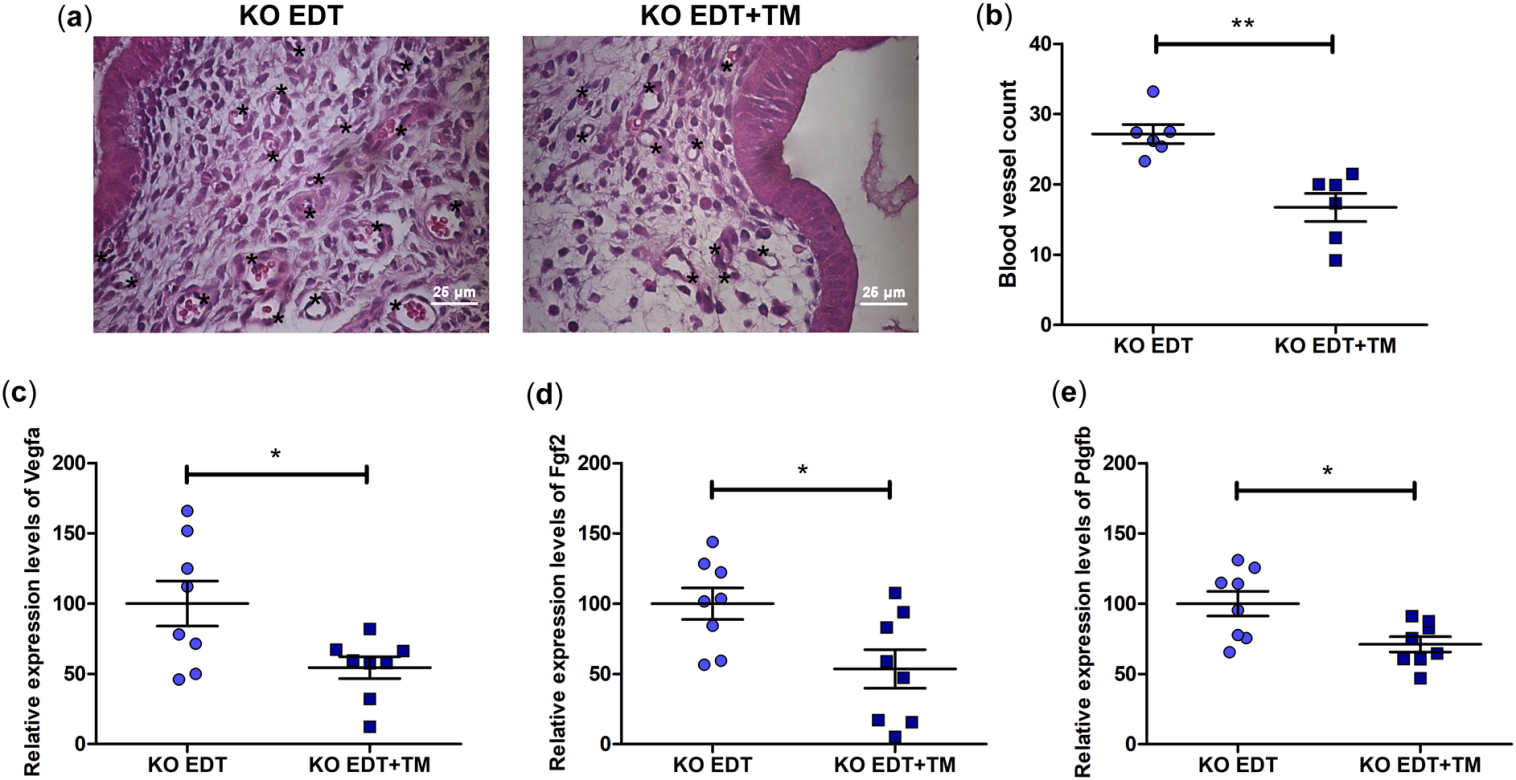
Effect of TM on the angiogenic process in endometriotic-like lesions in TNFR1^−/−^ mice. The micrographs show representative histological sections of endometriotic-like lesions induced in untreated (KO EDT) and TM-treated (KO EDT+TM) mice (**a**). Blood vessels of different calibers are marked with an asterisk. Magnification: 400 ×. Scale bar, 25 µm. The counting of blood vessels was performed on sections stained with hematoxylin–eosin using an optical light microscope for both experimental groups (**b**). Results are expressed as mean ± SEM (n = 6 animals/group). The mRNA expression of *Vegfa* (**c**), *Fgf2* (**d**), and *Pdgfb* (**e**) in endometriotic-like lesions was evaluated for both experimental groups. The relative quantification of each mRNA was calculated from the Cq values obtained for the genes of interest and the reference gene (*Rn18s*) using the 2^−ΔΔCt^ method. Results are expressed as mean ± SEM (n = 8 animals/group). KO EDT: circles; KO EDT+TM: squares. Statistical comparisons were made using Student's *t* test. *P < 0.05; **P < 0.01.

The importance of angiogenesis for the sustained development of endometriotic lesions is well known. Different studies suggest that TM can interfere with this process. Therefore, we evaluated its effect in our experimental EDT TNFR1-deficient model. On the one hand, we were able to observe in sections of endometriotic-like lesions stained with hematoxylin–eosin (Fig. 5a) that TM significantly decreased the number of blood vessels (*P* < 0.01; Fig. 5b). On the other hand, this Cu chelator also reduced the mRNA expression of *Vegfa* (*P* < 0.05; Fig. 5c), *Fgf2* (*P* < 0.05; Fig. 5d), and *Pdgfb* (*P* < 0.05; Fig. 5e) compared to the KO EDT group.

### TM decreased the SOD and CAT activity and increased the lipid peroxidation in endometriotic-like lesions of TNFR1-deficient mice

**Figure 6:**
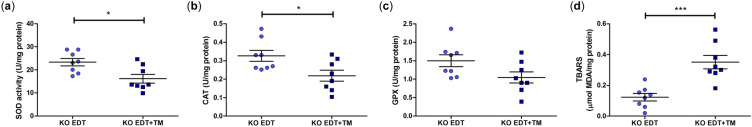
Effect of TM on oxidative stress in endometriotic-like lesions of TNFR1^−/−^ mice. The activity of the antioxidant enzymes SOD (**a**), CAT (**b**), and GPX (**c**) and the levels of MDA (**d**) were analyzed in lesions of untreated mice (KO EDT; circles) and TM-treated mice (KO EDT+TM; squares). Results are expressed as mean ± SEM (n = 8 animals/group). Statistical comparisons were made using Student's *t* test. *P < 0.05; ***P < 0.001.

Cu is an integral part of many important enzymes and is a redox-active metal. Therefore, we evaluate the effect of TM on antioxidant enzymatic defenses. We observed that this Cu chelator decreased the SOD activity (*P* < 0.05; Fig. 6a) and CAT activity (*P* < 0.05; Fig. 6b) but did not change GPX activity (Fig. 6c) in endometriotic-like lesions compared to the KO EDT group. In addition, we analyzed TBARS-MDA, a biomarker of lipid oxidation. TM significantly increased the MDA concentration in endometriotic-like lesions concerning the KO EDT group (*P* < 0.001; Fig. 6d), showing that this drug modifies the oxidative status.

## Discussion

In different studies, it has been observed that the TNF-α/TNFR system and Cu are involved in the EDT progression^3–6,15,16^. Therefore, in this work, we evaluated the therapeutic potential of Cu chelation by TM in a TNFR1^−/−^ worsened EDT status in mice. Under these experimental conditions, we found increased levels of Cu in the peritoneal fluid. TM reduced the levels of this metal to physiological concentrations without compromising animal welfare. This ‘old drug’ has been repositioned for cancer some years ago due to its anti-proliferative and anti-angiogenic effects in animal models and clinical studies^28,29^. Although EDT is not cancer in itself, it exhibits similar characteristics: progressive and invasive growth, recurrence, and a tendency to metastasize and develop its blood supply^30^, which prevail in animal models of the pathology and in conditions of imbalance of the TNF-α/TNFR system^5^. Based on this, we analyzed the effect of the administration of this drug in the experimental EDT induced in TNFR1^−/−^ mice.

Regarding the volume of the lesions, in TNFR1-deficient animals, the values obtained are higher than those previously reported in wild-type animals^26^, showing similarity with that reported by Vallcaneras et al.^5^. Therefore, our results continue to support that TNFR1 deficiency exacerbates EDT. According to Vallcaneras et al., this worsening of the pathology is due, among other factors, to a decrease in cell death and an increase in proliferation^5^. The observed increase in the size of the lesions is probably due to the activation of TNFR2-dependent pathways. The involvement of TNFR2 in various tumor cell types has been demonstrated^31^, promoting tumor growth through signal transduction cascades such as the Akt signaling pathway and the NF-κB induction through p42/p44 mitogen-activated protein kinase (MAPK)/ERK pathway^8^. In addition, blockade of TNFR2 has been shown to decrease tumor growth^32^ and EDT development^33^, which would indicate that this receptor is more necessary for the progression of proliferative diseases than the activation of p42/p44 MAPK/ERK and Akt through TNFR1. Therefore, we evaluated whether TM treatment affected *Tnfr2* mRNA expression. We observed this Cu chelator significantly reduced *Tnfr2* expression in endometriotic-like lesions (Supplementary Fig. S1). Importantly, TM treatment affected the volume and weight of lesions induced in TNFR1^−/−^ mice. This fact could be associated with the Cu levels decrease since this metal can stimulate the aforementioned signaling pathways^34,35^, among other effects, which define it as a factor that promotes tumor progression. Supporting all of the above, we found that this Cu chelator reduced cell proliferation rate compared to lesions in the KO EDT group.

Furthermore, we showed that in EDT-induced KO animals, the development of this disease increased estradiol levels in peritoneal fluid. It is known that Cu can modulate steroidogenesis: at low levels, it can decrease serum dehydroepiandrosterone (DHEA)^36^, a precursor of estradiol along with cholesterol, while at high levels, it can stimulate the expression of enzymes involved in estradiol synthesis^23^. Indeed, we determined that the Cu chelation with TM restored estradiol levels in the peritoneal fluid. We also demonstrated a positive correlation between estradiol levels and the volume of endometriotic-like lesions. This fact supports once again that EDT is an estrogen-dependent disease^1^. Interestingly, like TNFR2 and Cu, estradiol promotes tumor growth and cell proliferation^8,34,35,37^. In this sense, the TM action on Cu and estradiol levels in our experimental model, as well as its recognized ability to prevent the activation of signaling pathways that drive cell proliferation^38,39^, could justify its efficacy in reducing lesion development even in the absence of TNFR1.

Along with cell proliferation, the establishment of new blood vessels is essential for endometriotic lesions to develop^40^. We have previously observed anti-angiogenic effects of TM in EDT-induced wild-type mice, which hampered the progression of the pathology^26^. In the present study with TNFR1^−/−^ mice, we observed that treatment with TM reduced the number of blood vessels and the mRNA expression of the three pro-angiogenic factors analyzed (*Vegf, Fgf2*, and *Pdgfb*). It is argued that the combined inhibition of VEGF, FGF-2, and PDGF-B would be very effective in suppressing the vascularization of endometriotic lesions^41^; in fact, our results support the above. The decrease in the expression of the pro-angiogenic factors analyzed may be because TM inhibits the activation of NF-κB^28,42^. On the one hand, TM affects the phosphorylation and consequent degradation of IκBα, the major inhibitory protein of NF-κB^43^. On the other hand, TM decreases the expression of inhibitors of apoptosis proteins (IAP family)^44,45^, which are increased in EDT^46^ and are required for NF-κB activation through TNFR1 and TNFR2^47^. In our experimental TNFR1^−/−^ research design, we have shown that TM decreases the expression of *Tnfr2* mRNA. Taking all of the above into account, it is likely that TM has a strong inhibitory action on NF-κB in these deficient animals, compromising key downstream pathways for EDT progression. It is supported by the fact that, unlike what was observed in wild-type animals^26^, the Cu chelator administration, besides reducing the expression of *Fgf2* and *Pdgfb*, significantly reduced the *Vegfa* expression, being effective in this worsened state of the pathology. It is important to mention that, in the absence of TNFR1, all TNFR2-dependent pathways become relevant, particularly those related to cell proliferation, survival, and angiogenesis^8^. In fact, in a previous study carried out in TNFR1^−/−^ animals, it was found that there was greater activation of NF-κB than in wild-type animals^14^. Therefore, we postulate that, in the absence of TNFR1, there is not only an interruption of the recognized crosstalk between both TNFRs^10^ but also an alteration of TNFR2 signaling by TM. In addition to the above, TM has been shown to inhibit SOD1 enzyme activity, thereby affecting angiogenesis and inducing vessel abnormalities^48^, possibly by downregulating PDGFR^49^.

Deregulation of antioxidant defenses leads to the establishment of a pro-oxidant environment in proliferative diseases. In this regard, an increase in SOD activity and a decrease in CAT activity have been described in EDT, associated with an increase in the production of reactive oxygen species (ROS)^50^. Among the ROS is hydrogen peroxide, which acts as a second messenger in cell signaling to maintain an EDT-associated proliferative phenotype^51^. However, the overproduction of ROS can also promote cell damage. Delsouc et al. reported that in TNFR1^−/−^ mice with induced EDT, antioxidant protection increases in the peritoneal cavity with respect to the wild-type group, supporting the exacerbation of the disease^6^. Here we observe that TM administration decreases SOD and CAT activity, possibly tipping the balance towards ROS-induced apoptotic signaling. Our assumption is supported by the reduced volume of lesions and increased MDA concentration in these tissues, suggesting oxidative damage. While the increase in SOD activity has been postulated as a compensatory mechanism to control the increase in oxidative stress in EDT, its inhibition has been shown to induce an oxidative burst that produces cell and tissue damage^50^. The decrease in SOD activity could be due to the ability of TM to remove Cu ions from the active site of SOD1^52^. Moreover, TM could be indirectly responsible for the reduction of CAT activity by affecting the dismutation of the superoxide radicals to molecular oxygen and hydrogen peroxide; the latter is converted by CAT in molecular oxygen and water. This fact would reinforce the induction of the oxidative burst. SOD1 is more than an antioxidant enzyme; its inhibition by TM contributes to attenuating angiogenesis and tumor cell proliferation^48^. In fact, all of these processes were compromised in our TNFR1-deficient experimental model after the drug administration period, preventing EDT progression.

In summary, TM administration inhibits EDT progression in TNFR1^−/−^ mice, in which the pathology is aggravated. Given the loss of Cu homeostasis in this complex pathology, our preclinical evidence in a situation of imbalance in the TNF-α/TNFR system suggests that TM has therapeutic potential. This drug needs to continue to be evaluated in different EDT experimental research designs for possible repositioning.

## Methods

Two-month-old female TNFR1^−/−^ C57BL/6 mice, weighing 19–21 g, were used. Animals were housed in the Universidad Nacional de San Luis Animal Facility (San Luis, Argentina) under strict light conditions (12 h light, 12 h darkness), controlled temperature (22 ± 2 °C), sterile water, and ad libitum feeding. All experimental procedures were performed following the Guide for the Care and Use of Laboratory Animals of the National Research Council (8th ed., 2011, Washington, DC) and complied with the ARRIVE guidelines 2.0. This study was reviewed and approved by the Comité Institucional de Cuidado y Uso de Animales (CICUA) of the Universidad Nacional de San Luis (Protocols No. B-304/20 and B-304/21).

### Experimental design

Twenty-four TNFR1^−/−^ mice (KO) were randomly divided into three groups: (1) sham-operated mice (KO Sham), (2) EDT-induced mice (KO EDT), and (3) TM-treated EDT-induced mice (KO EDT+TM). The EDT induction consisted of autologous uterine tissue transplantation to the intestinal mesentery^5,26^. For this, animals were anesthetized with an intraperitoneal injection of ketamine-xylazine: 100 mg/kg of ketamine and 10 mg/kg of xylazine (Holliday Scott, Buenos Aires, Argentina, and Richmond, Buenos Aires, Argentina, respectively). After a mid-ventral incision, the right uterine horn was removed from the animal, divided longitudinally, and then cut into four mm^2^ pieces. The fragments were sutured in the intestinal mesentery with a 6-0 nylon suture to simulate endometriotic lesions. In Sham mice, three sutures were made without implanting uterine tissue. The mice's body weight, food consumption, and grooming behavior were monitored daily. One month after inducing EDT, animals were euthanized by cervical dislocation. Immediately, a small mid-ventral orifice was opened, through which 1.5 mL of pH 7.4 phosphate-buffered saline (PBS) was injected into the peritoneal cavity of each animal. Peritoneal lavage fluid was collected and centrifuged at 250 g for 10 min at 4 °C. Supernatants (*peritoneal fluid*) were collected and stored at − 80 °C for Cu and estradiol determination. Finally, the abdomen of the EDT-induced animals was fully opened to access the endometriotic-like lesions.

### Administration of TM

Starting on postoperative day 15 (period required for lesion establishment), each animal in the KO EDT+TM group received 0.3 mg of TM (cat# 323446, Sigma-Aldrich, St Louis, MO, USA) orally, as previously described^26^. Weekly controls of body weight and hematocrit were performed to ensure that severe Cu deficiency did not occur^42,53^. Furthermore, no toxicity evidence was observed for the administered dose based on food consumption or grooming behavior compared to the KO Sham and KO EDT groups.

### Cu determination

Cu concentration in the peritoneal fluid was determined by electrothermal atomic absorption spectrometry (ETAAS) with a graphite furnace. Peritoneal fluid samples (500 μL) were mineralized with 500 μL concentrated nitric acid and 500 μL hydrogen peroxide. After adding the solutions, transparent samples were obtained by heating at 60 °C for 1 h in a thermostated water bath. All samples and reagents were prepared in 15 mL metal-free polypropylene tubes (Sarstedt, Germany). The reagents used were of trace analysis grade. They included: Ultrapure water with a resistivity of 18.2 MΩ cm produced by an Easy pure RF system from Barnstead (Dubuque, IA, USA), Double distilled acids obtained with a PTFE sub-boiling acid distiller (Distillacid, Berghof Products+Instruments GmbH, Germany) and 30% hydrogen peroxide (Merck, Germany). A Shimadzu Model AA-7000 atomic absorption spectrometer (Tokyo, Japan) was used to perform the measurements, equipped with a GFA-EX7 atomizer and an ASC-6100 autosampler. Integrated platform graphite tubes (L'vov), Shimadzu (Tokyo, Japan), were used in all experiments. A Cu hollow cathode lamp (Hamamatsu, Photonics, K.K., Japan) was employed as a radiation source at 324.8 nm with a 0.5 nm slit. All measurements were performed in duplicate. The results were expressed in µg/L.

### Macroscopic analysis of the endometriotic-like lesions

Lesions were identified, counted, and measured with a caliper at two different perpendicular diameters. For the volume calculation, the following equation was used: V = (4/3) π r_1_^2^ r_2_ (r_1_ and r_2_ are the radiuses and r_1_ < r_2_). Subsequently, the lesions were removed and weighed. For each animal, a lesion was fixed in 4% paraformaldehyde in PBS (pH 7.4) for 24 h at 4 °C. Fixed specimens were embedded in paraffin and cut into 4-μm-thick sections. A standard hematoxylin-eosin procedure was used to confirm the presence of endometrial glands and stroma in the ectopic tissue and to count the number of blood vessels. Other 4-μm-sections were prepared for cell proliferation study. Another lesion was placed at − 20 °C in RNAhold^®^ (TransGen Biotech^®^ Co., Ltd., Beijing, China) for RT-qPCR studies. The third lesion was kept at − 80 °C for protein extraction.

### Estradiol determination

An electrochemiluminescence immunoassay (ECLIA) kit specific for estradiol (Elecsys Estradiol III, Roche Diagnostics International Ltd., Mannheim, Germany) was used, according to manufacturer's instructions. The lower and upper detection limits were 5 and 3000 pg/mL, respectively. All measurements were performed in duplicate. Estradiol levels in the peritoneal fluid were expressed in pg/mL.

### Immunohistochemistry of PCNA

Proliferating cell nuclear antigen (PCNA) is a nuclear protein involved in cellular DNA replication; therefore, it was evaluated in endometriotic-like lesions of untreated and TM-treated mice by immunohistochemistry. Tissue sections from six different animals per experimental group were deparaffinized in xylene and rehydrated in a graded series of ethyl alcohols. For antigen retrieval, slides were transferred to a glass staining jar filled with 0.01 M sodium citrate buffer (pH 6.0), which was placed in a microwave oven for 10 min on the highest power. Endogenous peroxidase was blocked with 3% H_2_O_2_ for 30 min. All the sections were blocked with 4% BSA in 1 × PBS for 2 h at room temperature in a humid chamber and then incubated with a polyclonal rabbit anti-PCNA antibody (1:150; FL-261, Santa Cruz Biotechnology, CA, USA) overnight at 4 °C. For negative controls, the primary antibody was replaced with 1% BSA in 1 × PBS. Subsequently, sections were incubated with a biotinylated goat anti-rabbit IgG antibody (1:500; B8895, Sigma-Aldrich, St Louis, MO, USA) for 1 h at room temperature. Then, they were incubated with HRP-conjugated streptavidin (VectorLabs, Burlingame, CA, USA) for 30 min at room temperature. Diaminobenzidine (DAB) was used as substrate and incubated for 5 min (Cell Marque, CA, USA). Finally, tissue sections were counterstained with hematoxylin, dehydrated with graded alcohols, cleared in xylene, and properly mounted. PCNA-positive cells were identified by the presence of brown nuclear reactivity. Their percentage was established by two different operators at 1000 × under optical light microscopy (Olympus, Japan) by analyzing six representative fields per section. The total positive cell percentage was calculated per slide and was used to obtain the mean of each experimental group.

### Mean blood vessel count

Blood vessels of different calibers were counted in sections of endometriotic-like lesions stained with hematoxylin-eosin using an optical light microscope (Olympus, Japan) at 400 ×. Tissue sections from six different animals per experimental group and between 6 and 8 fields per section were observed by two different operators. Blood vessels identified in each section were reviewed and approved by a pathologist. Results were expressed as the mean blood vessel count per field for the KO EDT and KO EDT+TM groups.

### Quantitative reverse transcription PCR (RT-qPCR)

RT-qPCR was performed to analyze the gene expression of the following genes: *Vegfa*, *Fgf2*, and Platelet-Derived Growth Factor B (*Pdgfb*). Total RNA was isolated from endometriotic-like lesions using TRIzol^®^ reagent (Thermo Fisher Scientific, Inc., Waltham, MA, USA). RNA samples were quantified using an EPOCH™ microplate spectrophotometer (BioTek Instruments, Inc., Vermont, USA). All high-purity and intact RNA samples were treated with RQ1 RNase-Free DNase (Promega). Total RNA (1 μg) was reverse-transcribed using Transcriptor First Strand cDNA Synthesis Kit (Roche Diagnostics International Ltd., Mannheim, Germany) and stored at − 20 °C, following manufacturer's guidelines. For qPCR, cDNA was amplified in an ABI PRISM^®^ 7500 Instrument (Applied Biosystems, USA), using FastStart™ Universal SYBR^®^ Green Master (Roche Diagnostics International Ltd., Mannheim, Germany). The reaction mixture consisted of 2×FastStart™ Universal SYBR^®^ Green Master Mix, cDNA, forward primer (10 μM), reverse primer (10 μM), and nuclease-free water. All primers are described in Table 1. PCR cycling conditions were 95 °C, 10 min; 40 cycles at 95 °C, 15 s; 60 °C, 1 min. The relative expression was calculated using the 2^−ΔΔCt^ method^54^. All experiments were performed in duplicate. *Rn18s* (18S ribosomal RNA): internal reference gene.

**Table 1:** Primer gene symbols, sequences, GenBank access numbers, and sizes of amplicons.

Gene	Sequences (5′–3′)	GenBank access number	Amplicon (bp)
*Vegfa*	Forward: CACTTCCAGAAACACGACAAAC	NM_001025250.3	95
	Reverse: TGGAACCGGCATCTTTATCTC		
*Fgf2*	Forward: GGCATCACCTCGCTTCC	NM_008006.2	97
	Reverse: CGCCGTTCTTGCAGTAGAG		
*Pdgfb*	Forward: GAGTGTGGGCAGGGTTATTT	NM_011057.4	105
	Reverse: GAATCAGGCATCGAGACAGAC		
*Rn18s*	Forward: CTGAGAAACGGCTACCACATC	NR_003278.3	107
	Reverse: GCCTCGAAAGAGTCCTGTATTG		

### Antioxidant enzyme activities

Proteins from lesions were extracted in 100 μL RIPA buffer (Thermo Fisher Scientific Inc., Waltham, MA, USA) and quantified according to Bradford method^55^, as previously described^26^. To measure superoxide dismutase (SOD) activity, the pyrogallol autoxidation method was used, monitoring the change in absorbance at 420 nm per min. One unit of the enzyme was expressed as the amount of SOD that inhibits 50% of pyrogallol autoxidation^56^. The catalase (CAT) activity was determined by measuring the decrease in H_2_O_2_ absorption at 240 nm, in which one CAT unit is the amount of enzyme required to decompose 1 μM of H_2_O_2_/min^57^. The glutathione peroxidase (GPX) activity was determined by following NADPH oxidation at 340 nm^58^. All readings were carried out using a Shimadzu 1800 UV–Visible spectrophotometer. The results were expressed as units of enzyme activity per milligram of protein (U/mg protein). All measurements were performed in duplicate.

### Measurement of MDA

The levels of malondialdehyde (MDA), the end product of lipid peroxidation, were determined according to the method described by Draper and Hadley^59^. This method was based on the reaction of MDA with thiobarbituric acid (TBA). 1,1,3,3-tetraethoxypropane was used as a standard for the calibration curve, and the results were expressed as μmol MDA/mg protein. All measurements were performed in duplicate.

### Statistical analysis

Statistical analysis was performed using GraphPad Prism 5.0 software (GraphPad Software Inc., San Diego, CA, USA). Values were presented as the mean ± SEM. Differences between groups were analyzed using a two-tailed unpaired Student’s *t* test or one-way ANOVA followed by Tukey’s multiple comparison test (when appropriate). For the correlation analysis, the normality of data was assessed with the Shapiro–Wilk test, and Spearman’s correlation method was applied. Differences were statistically significant when *P* < 0.05.

## Supplementary Information

Supplementary Figure S1.

## Data Availability

Data used to support the findings of this study are available upon request from the corresponding authors (M.C. and M.B.D.).
