# Supplementary material for: The copper chelator ammonium tetrathiomolybdate inhibits the progression of experimental endometriosis in TNFR1-deficient mice
Source: Sci Rep. 2023 Jun 26;13:10354. doi: 10.1038/s41598-023-37031-1 (PMC10293193; doi:10.1038/s41598-023-37031-1)
Supplement: Supplementary file 1 — Supplementary Figure S1. [file 41598_2023_37031_MOESM1_ESM.pdf]

The copper chelator ammonium tetrathiomolybdate inhibits the progression of experimental endometriosis in TNFR1-deficient mice

Rocío Ayelem Conforti[1], María Belén Delsouc[1,*], Ana Sofia Zabala[1], Sandra Silvina Vallcaneras[1], and Marilina Casais[1,*]

[1]Facultad de Química, Bioquímica y Farmacia, Universidad Nacional de San Luis (UNSL). Instituto Multidisciplinario de Investigaciones Biológicas de San Luis (IMIBIO-SL-CONICET), D5700HHW, San Luis, Argentina.
[*]mcasais@unsl.edu.ar (M.C.); mbdelsouc@unsl.edu.ar (M.B.D.)

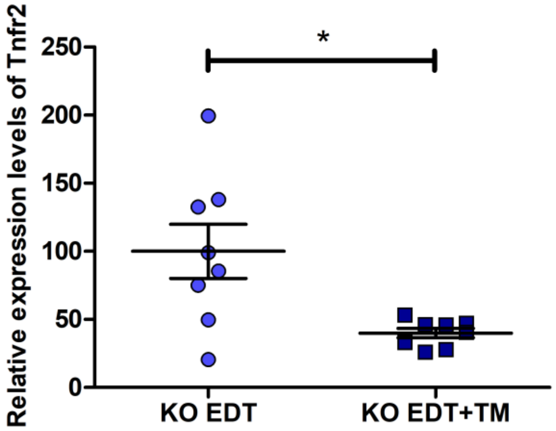

Supplementary Figure S1. Evaluation of *Tnfr2* expression in endometriotic-like lesions induced in TNFR1$^{-/-}$ mice. The mRNA expression of *Tnfr2* was evaluated in untreated mice (KO EDT, circles) and TM-treated mice (KO EDT+TM, squares) by RT-qPCR. We observed that treatment with TM significantly reduced the *Tnfr2* mRNA expression in endometriotic-like lesions in TNFR1$^{-/-}$ mice. The relative quantification of mRNA was calculated from the Cq values obtained for the gene of interest and the reference gene (*Rn18s*) using the $2^{-\Delta\Delta Ct}$ method (Livak, K. J. & Schmittgen, T. D., 2001). The primers used for the *Tnfr2* amplification (GenBank access number: NM_011610.3) were GTCTGGAACCAGTTTCGTACAT (*Forward*) and ACACTCGGTTCTGCTGTTTAG (*Reverse*). Amplicon size: 97 bp. PCR cycling conditions were 95 °C, 10 min; 40 cycles at 95 °C, 15 s; 60 °C, 1 min. Results are expressed as mean ± SEM (n = 8 animals/group). All experiments were performed in duplicate. Statistical comparisons were made using Student's t-test. *$P < 0.05$
